# Supplementary material for: Venom gland organogenesis in the common house spider
Source: Sci Rep. 2024 Jul 4;14:15379. doi: 10.1038/s41598-024-65336-2 (PMC11224297; doi:10.1038/s41598-024-65336-2)
Supplement: Supplementary file 4 — Supplementary Information. [file 41598_2024_65336_MOESM4_ESM.docx]

**Table S1. Details of the Leica Stellaris 5 White Light Laser inverted confocal microscope used to acquire the z-stack images.**

| **Stand** | - Leica DMi 8 (inverted microscope) |
| --- | --- |
| **Illumination** | - – |
| **Lasers** | - 405 nm Diode - Pulsed White Laser Line (WLL) – Tunable between 485 nm and 790 nm |
| **Objectives** | - HC PL APO CS2 10x 0.4 Dry - HC PL APO CS2 20x 0.75 Dry - HC PL APO CS2 40x 1.30 Oil - HC PL APO CS2 63x 1.40 Oil |
| **Stage** | - Motorized XY - Galvo Z-piezo |
| **Contrast** | - – |
| **CO2 \| T° control** | - Yes, stage-top incubation (Oko Lab) CO2, Temp. and Humidity control |
| **Detector type** | - 4 HyDs Highly sensitive detectors – Detection from visible to IR wavelengths on the 4 channels - Transmitted PMT |
| **Optional modules** | - FRAP/FRET wizard - Lightning (deconvolution) - Navigator (Tiling/stitching over huge areas) - TauSense (TauContrast, Gating, Separation and Interaction) |
| **Software** | - LAS X (2022) |


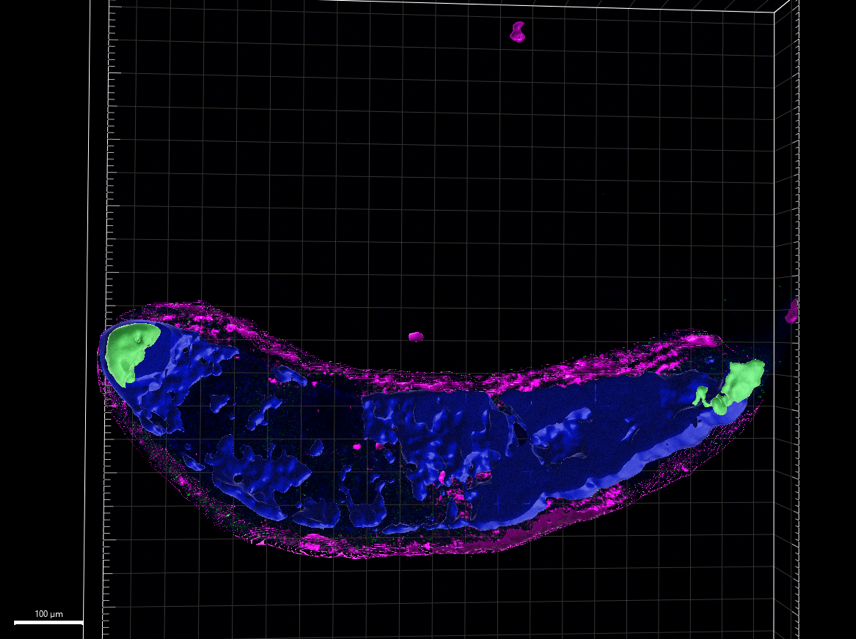

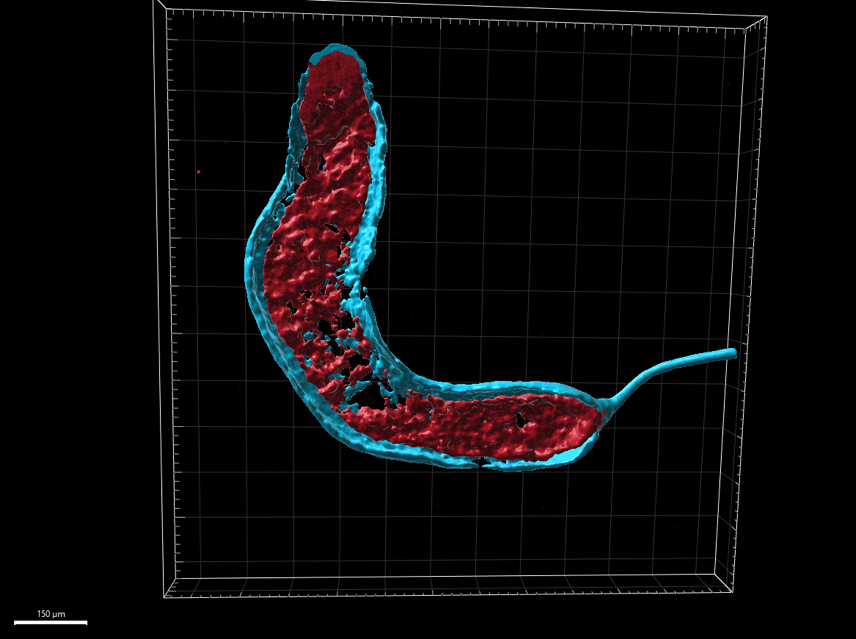


a

b

**Figure S1. 3D reconstruction of the *in situ* HCR signals of venom gland markers.** a) Expression of toxin (blue), *Dll* (green), and *sum-1* (magenta). Scale bar: 100 *μm*. b) Expression of *fkh* (cyan) and *sage* (red). Scale bar: 150 *μm*.


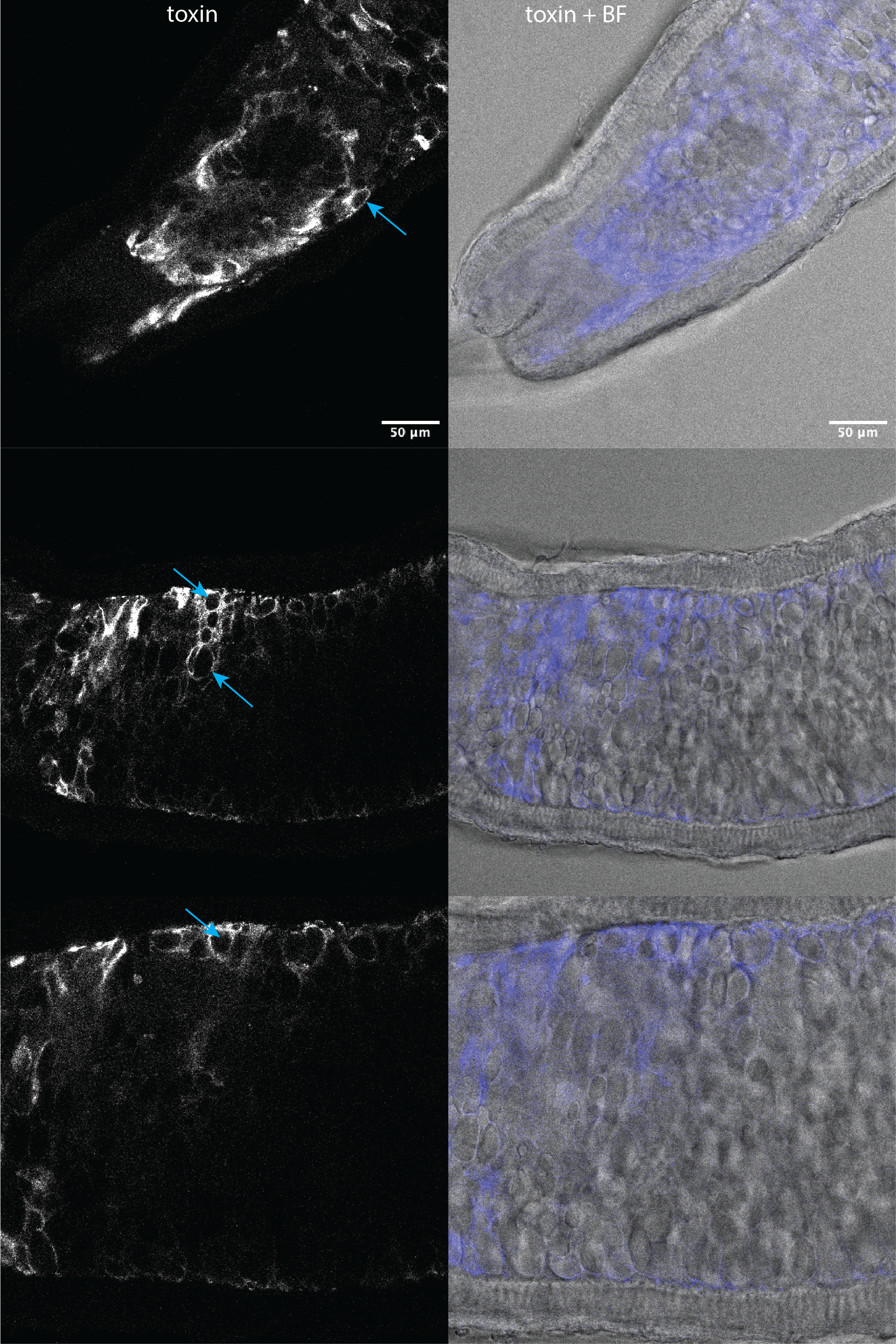


**Figure S2.** **Toxin gene expression in the adult venom gland.** Note the expression of toxin contouring the large secretory vesicles (light blue arrows). BF = brightfield. 63x oil.


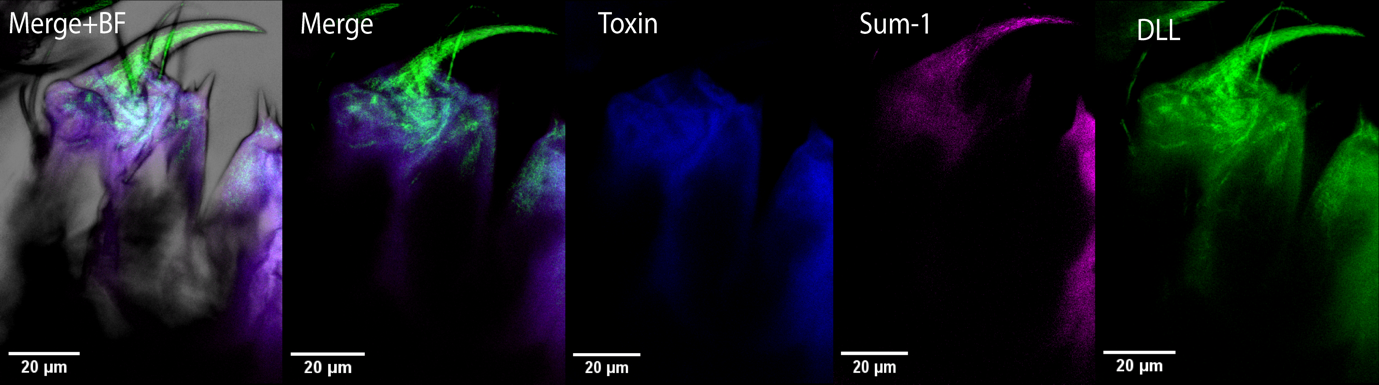


*toxin*

*Dll*

*sum-1*

**Figure S3. Expression of the toxin, *sum-1* and *Dll* markers at the tip of the chelicera of whole-mount first instar early stage.** *Dll* is expressed within the fangs together with *sum-1*, while the toxin gene is not expressed. BF = brightfield. 40x oil.

**BF**

*Dll*

*sum-1*

*sum-1*

**Figure S4. Expression of *sum-1* and *Dll* at stage 14.** Note the expression of *sum-1* in the coxa of the first leg and the log joints (arrow heads), along with expression in the venom glands (arrows) which are located at the base of the chelicerae. 20x dry.


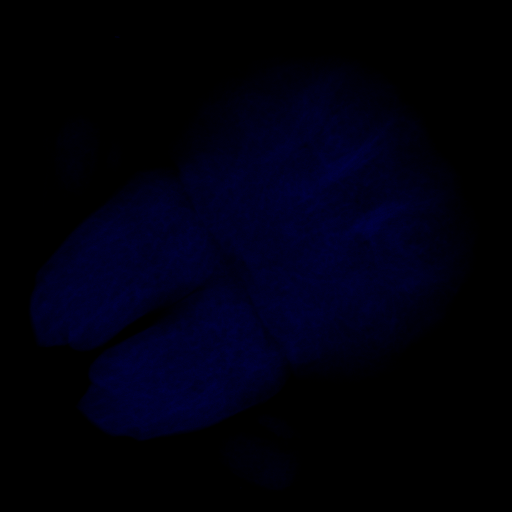

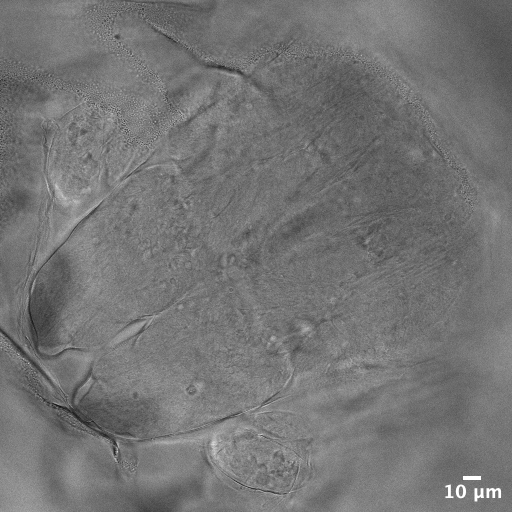


BF

toxin

**Figure S5. Expression of the toxin marker in embryos stage 14.** Toxin expression was not detected in the chelicerae nor in the prosoma at this stage. BF = brightfield. 40x oil.

**
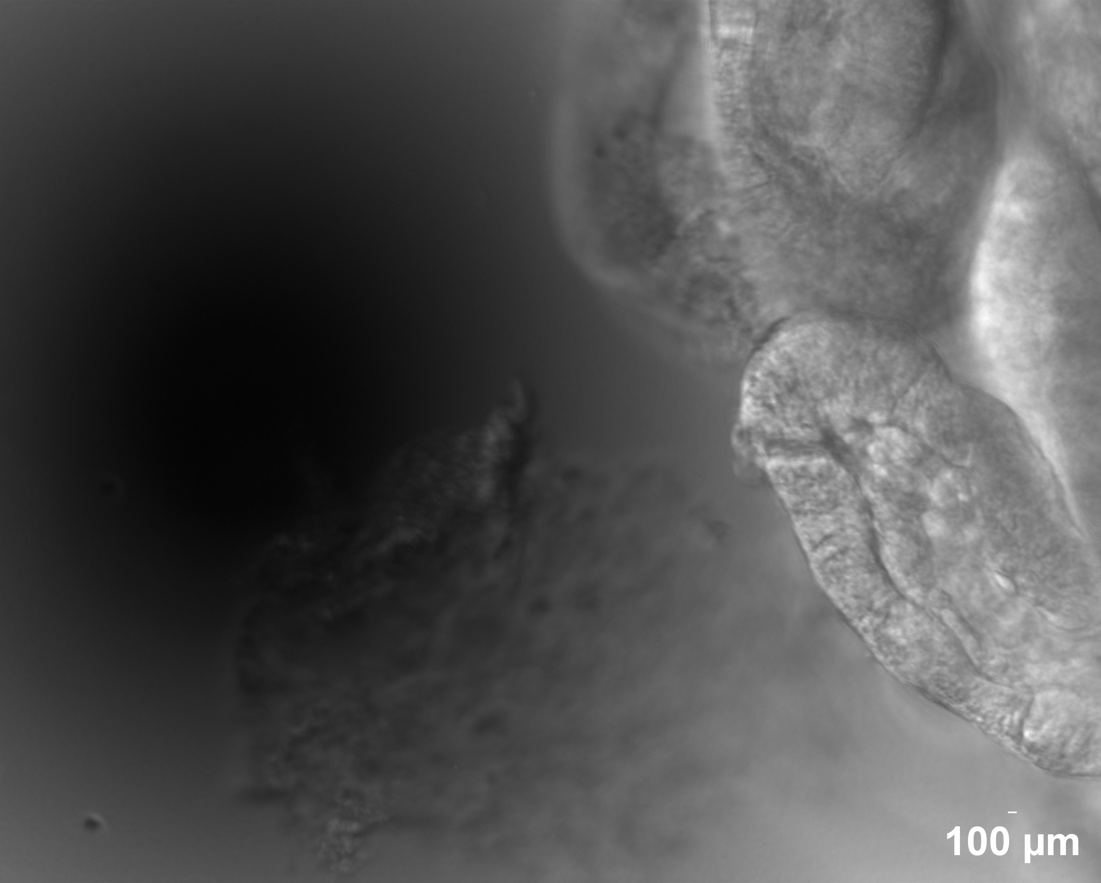

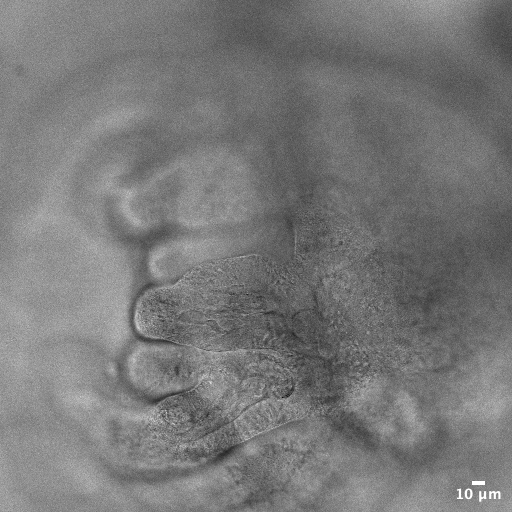
**

BF

DAPI

**Figure S6. Interior of the chelicerae of stage 13 embryos.** A lumen (arrow) is visible in the distal side of the chelicerae when stained with DAPI. An invagination (arrow) is visible inside the chelicerae in brightfield (BF) confocal laser scanning microscopy scan. The images are from two different individuals. 40x oil.

Merge

*Dll*

*sum-1*


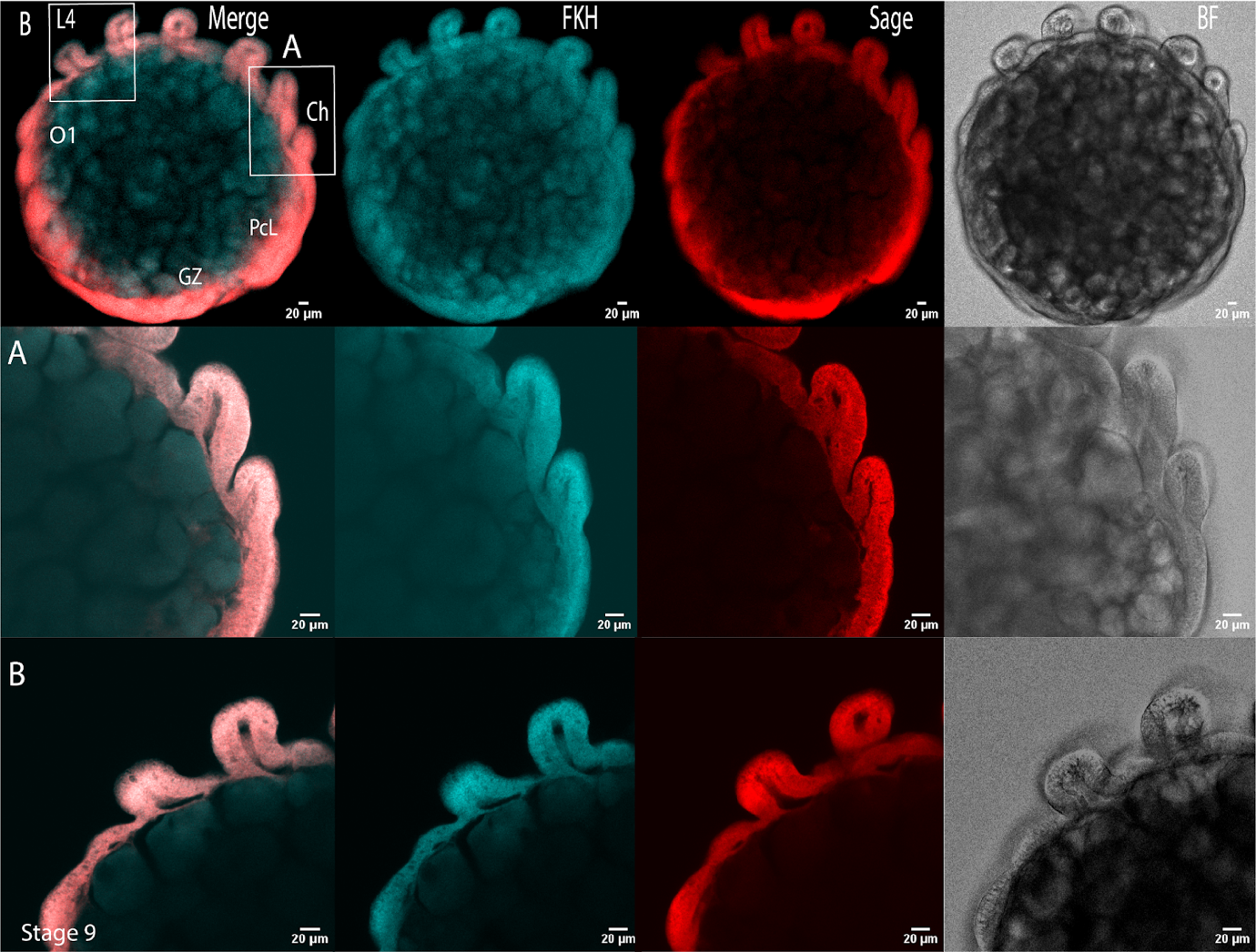


Ch

L4

*sage*

*fkh*

**Figure S7. Expression of marker genes in stage 9 embryos.** The chelicerae are still small buds, and the venom gland primordium has not emerged yet. All images 20x dry, except zoom-in of Ch and L4 with 40x oil. Ch = Chelicerae, GZ = Growth zone, L4 = fourth leg, O1 = First opisthosomal segment, PcL = Precheliceral lobe.


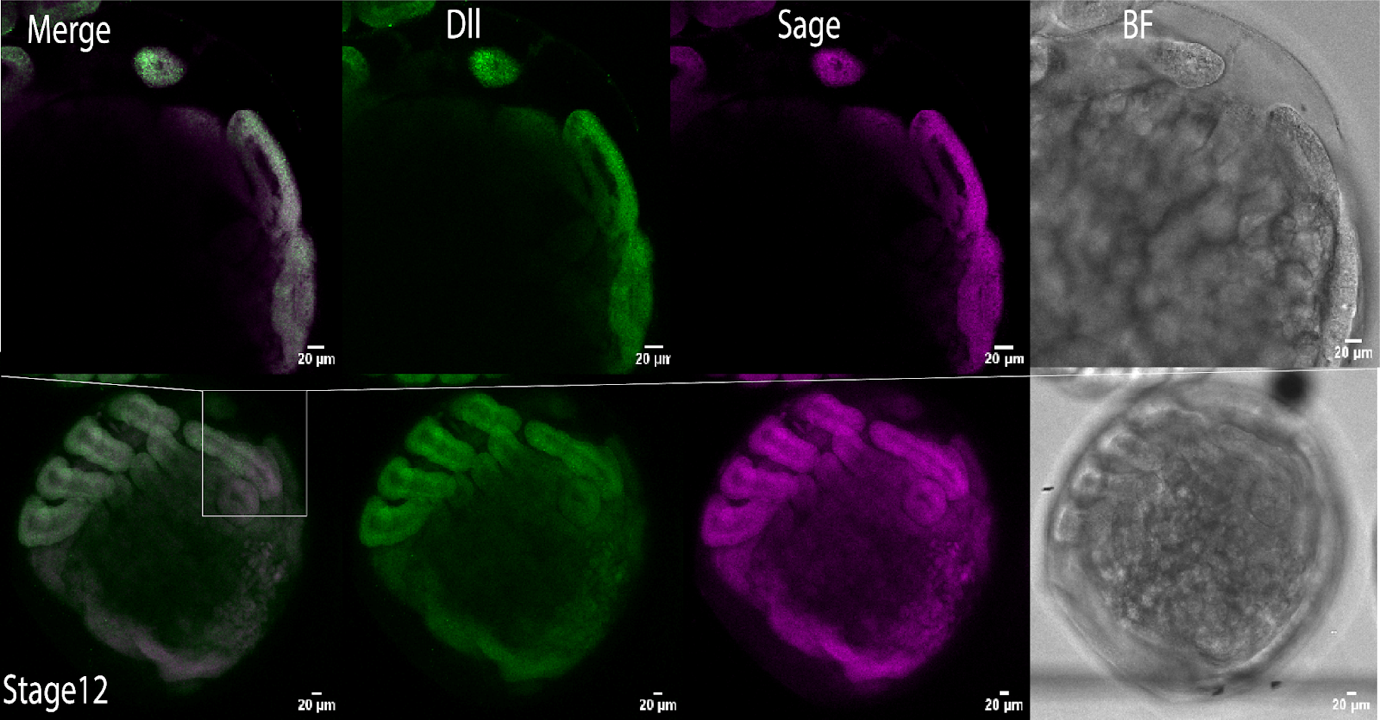


*Dll*

*sum-1*


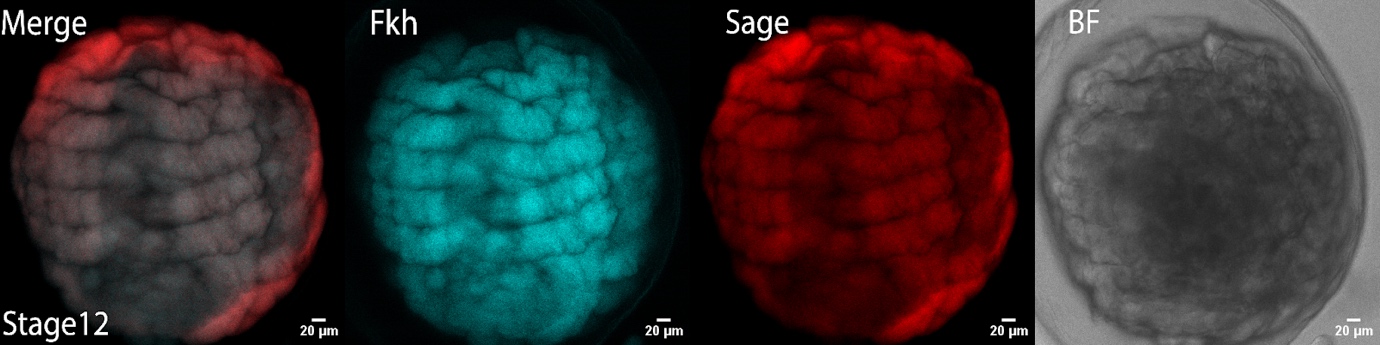


*fkh*

*sage*

**Figure S8. Expression of marker genes in stage 12 embryos.** At this stage, *Dll* and *sum-1* are expressed on the dorsal side of the chelicerae (arrows). The venom gland primordium has not emerged yet. 20x dry except first row with 40x oil.

**
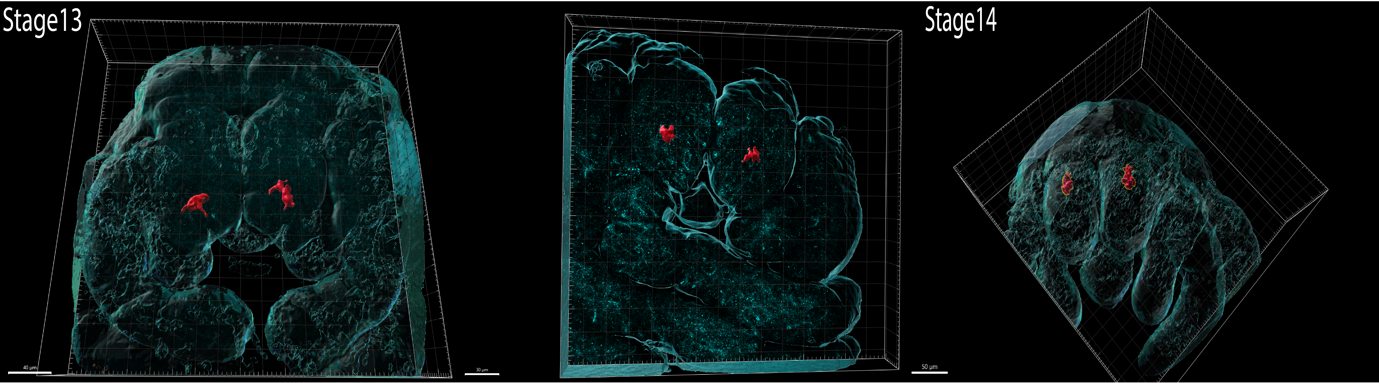
**

**Figure S9. 3D reconstruction of *sage* expression in embryos.** The venom gland primordium appears at the tip of the chelicerae at stage 13, and during the last embryonic stage it progresses toward the base of the chelicerae.


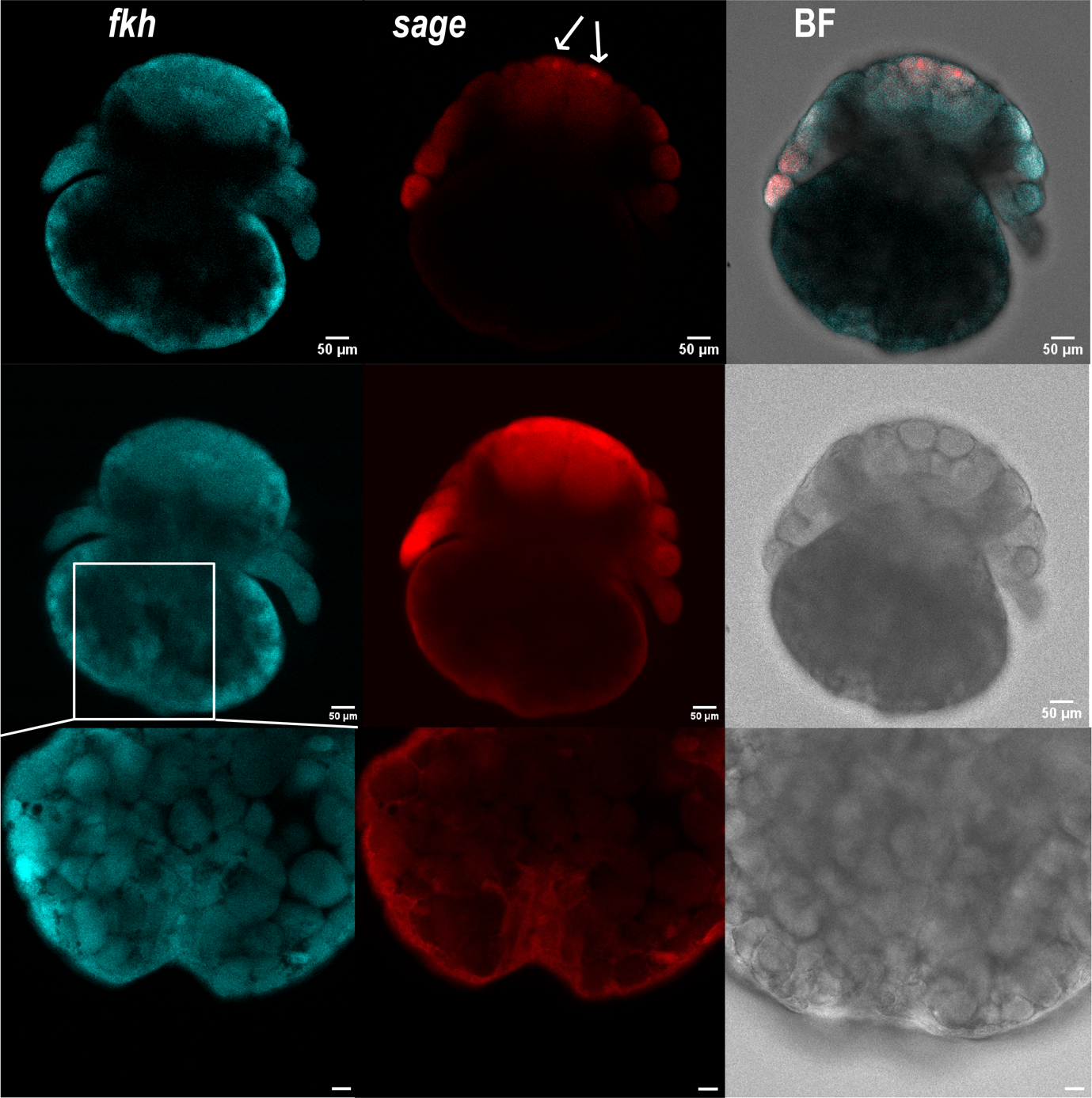


a

BF

+ Merge

bB

**Figure S10. Expression of *sage* and *fkh* in stage 14 embryos.** a) Focal plane at the chelicerae to detect expression in the venom glands. b) Focal plane on the ventral side of the opisthosoma in the area where the silk glands are thought to develop and zoom-in (rectangle). No signal corresponding to the silk glands was detected. 20x dry except zoom-in with 40x oil and scale bar 20 μm. BF = brightfield.


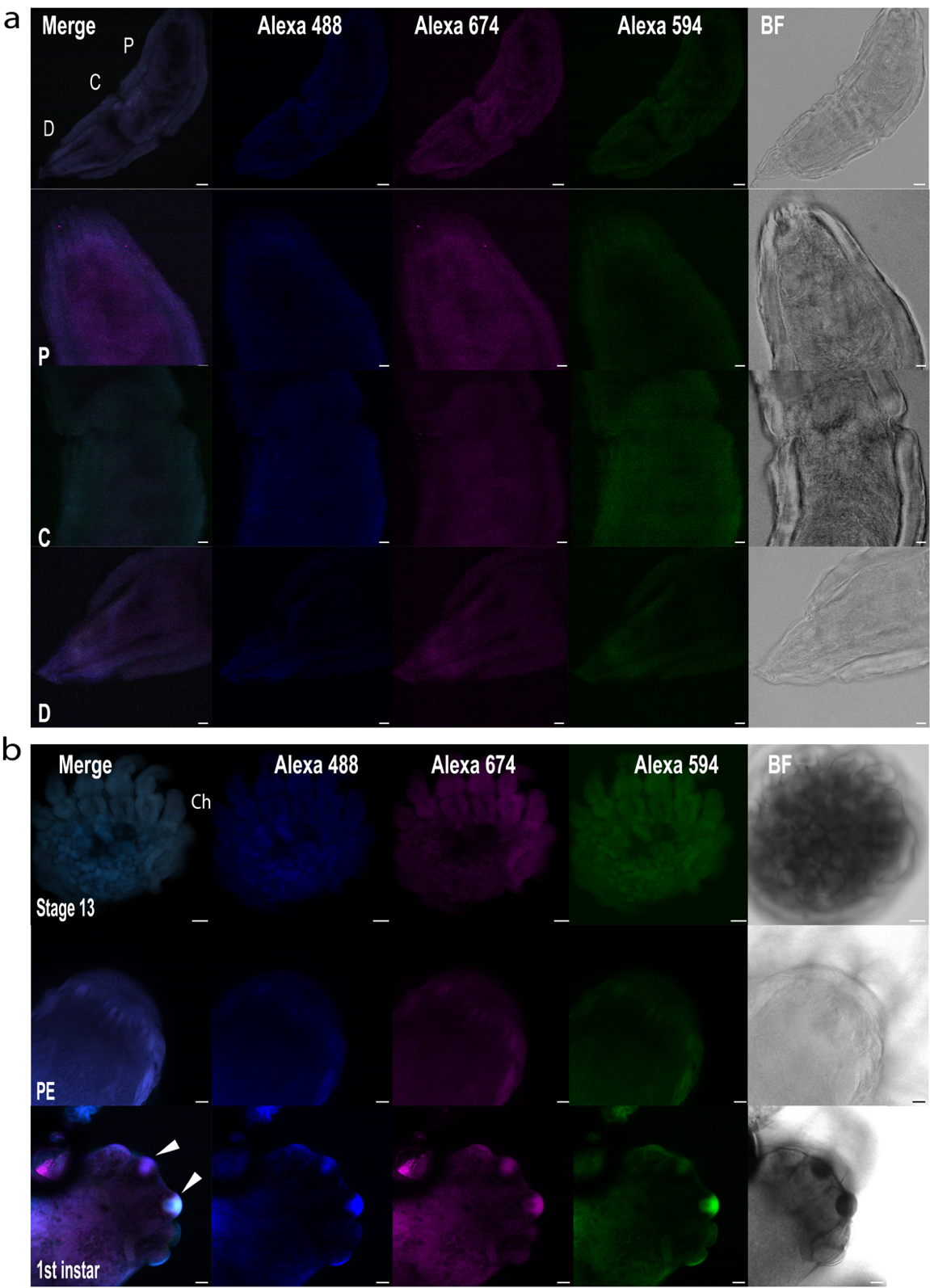


**Figure S11. Negative controls.** Experiments without the probes but with hairpins carrying the Alexa 488, 674, or 594 fluorescent dyes. a) Negative control of dissected whole-mount adult venom gland with zoom-in of the proximal (P), central (C), and distal (D) regions. No autofluorescence was observed. First row 20x dry and scale bar 50 μm, rows below 40x oil and scale bar: 20μm. b) Negative controls for whole mount embryo, postembryo (PE), and first (1^st^) instar stages. In the post eclosion stages, especially first instars, the eyes are highly autofluorescent (arrowheads). First row 20x dry and scale bar 50 μm, rows below 40x oil and scale bar: 20μm. BF = Brightfield, Ch = Chelicerae.
